# The ecological system’s influence on physical activities of older adults: comparison between older men and women

**DOI:** 10.1007/s40520-024-02908-2

**Published:** 2024-12-27

**Authors:** Su Yeon Roh, Ik Young Chang

**Affiliations:** 1 Department of Exercise Rehabilitation and Welfare, College of Health Science, Gachon University, Inchon, 21936 Republic of Korea; 2 Department of Sport Coaching, College of Sport Science, Korea National Sport University, Seoul, 05541 Republic of Korea

**Keywords:** Older adults, Physical activity, Ecological systems, South Korea

## Abstract

**Background:**

Korea is expected to become a super-aged society by 2025. Research has shown that regular participation in physical activity has a positive impact on older adults’ health and reduces national health costs.

**Aims:**

Drawing on Bronfenbrenner’s ecological model, this study examines ecological systems that influence physical activity in older men and women.

**Method:**

The data analysis included information on 537 older adults aged 65 years and older residing in South Korea. The regression analysis identified differences in the ecological systems that influence physical activity in older adults by sex.

**Results:**

By examining the ecological systems that affect physical activity in older men, this study found that the macrosystem affected the exosystem (*p* <.001) and microsystem (*p* <.001), the exosystem affected the mesosystem (*p* <.01), and the microsystem affected individuals (*p* <.001). In the case of older women, the macrosystem affected the exosystem (*p* <.001), microsystem (*p* <.001), and individuals (*p* <.01); the exosystem affected the microsystem (*p* <.01), and the microsystem affected individuals (*p* <.001).

**Conclusions:**

The microsystem commonly affects the physical activity of older men and women, whereas the macrosystem only affects the physical activity of older women, suggesting that older men may experience limited macrosystem support in fostering their participation in physical activity. To address this disparity, the study highlights the need for targeted policies to enhance macrosystem support for older men, such as developing tailored physical activity programs that promote positive attitudes and accessible opportunities for participation.

## Introduction

In 2018, South Korea (hereafter “Korea”) became an aging society. As of 2023, people aged 65 years or older (hereafter “older adults”) are approximately 18.4% of the total population (51,558,030). Korea is expected to become a super-aged society by 2025, with older adults accounting for 20.6% of the population [1].

Such a dramatic change in Korea’s demographic structure has sparked greater interest in the healthy lives of older people. On an individual level, older adults’ health significantly impacts their quality of life and happiness. Healthy people can enjoy free and abundant leisure time without pain due to a healthy lifestyle. At a societal level, the health of older adults is closely related to national finances. According to the 2022 National Health Insurance Statistical Yearbook [2], the number of people aged 65 years and older covered by health insurance in 2022 rose to 8.75 million, accounting for 17.0% of the total population. Consequently, total medical expenses for older adults also increased 1.4 times that in 2018, reaching US$ 333.95 billion in 2022.

Physical activity is recommended to improve the health of older adults. Regular exercise not only benefits their health but also significantly reduces nation’s medical costs [3]. However, two out of three Korean older adults do not meet the World Health Organization’s aerobic physical activity guidelines, and four out of five do not follow the strength exercise guidelines [4, 5].

Older men’s physical activity participation status is lower than that of their female counterparts. According to the 2023 Korea National Sports Participation Survey (KNSPS), 17.6% of older men do not engage in physical activities, compared to 15.2% of older women, reflecting a non-participation gap of 2.6% [3]. Conversely, the proportion of older women actively participating in physical activities stands at 11.1%, 5.4% higher than the 5.7% participation rate among older men. These findings highlight the lower physical activity engagement among older men compared to older women.

Examining the differences in physical activity levels between older men and women is essential because older adults’ aging and progression differ depending on sex. According to a study by Heo [6], men have poorer health on average than women. Specifically, aging becomes noticeable mainly around 50–55 years for men and 70–75 years for women, and men show faster aging than women until the age of 65 years [5]. In other words, because the aging characteristics of men and women differ depending on the stage of life, physical activity levels and factors affecting them should be analyzed differently [7, 8]. Recently, it has been argued that gender-specific strategies should be developed to increase physical activity participation rates [9]. Studying the aging characteristics of older people is to avoid viewing them as a single group. In other words, factors such as sex, age group, health status, and social stratification based on economic status must be carefully considered.

Bronfenbrenner’s ecological model has been widely used to analyze systems affecting older adults’ physical activity [10–15]. This model suggests that human development is influenced by interconnected environmental systems: the microsystem (close relationships like family), mesosystem (interactions between microsystems, e.g., family and work), exosystem (broader social structures like policies), and macrosystem (cultural ideologies like gender norms) [15].

Previous studies have examined the physical activity of older people as a single group without distinguishing important characteristics such as gender, using Bronfenbrenner’s ecological model, which can explain everything from personal to social domains. However, little research has been conducted to empirically analyze whether there are differences in ecological systems that affect physical activity between older men and women.

Therefore, this research aims to examine ecological systems influencing the physical activity of older males and females differently, drawing upon Bronfenbrenner’s ecological model. Following a short introduction, the research is divided into five sections. First, the paper provides a brief outline of Bronfenbrenner’s ecological model and reviews previous research that applied the model. Second, we outline the quantitative method used. Third, analyzing collected data, we look at the differences in ecological systems influencing physical activity between older men and women. Fourth, we discuss the analysis within the socio-cultural context, and finally, we offer a conclusion and recommendations for future research.

## Theoretical framework: Bronfenbrenner’s ecological model and physical activity of older adults

Bronfenbrenner’s ecological model explains how human development and behavior are influenced by interactions between systems, such as psychological, family, cultural, socioeconomic, and political domains, which ultimately shape our behavior, life decisions, and wellness over a lifetime. In other words, this model of human development connects disparate research fields to explain how individuals and the interplay of their environments contribute to their development [16, 17]. This model asserts that human development is an evolving complex reciprocal interaction frequently occurring over time between individuals, objects, and symbols in their environments. These environmental interactions are referred to as proximal processes, found when one learns new skills or performs complex tasks. Proximal processes aim to explain how individual characteristics and the immediate and distal environments in which the processes unfold result in desired or undesired developmental outcomes. For example, when individuals access and use technology to improve their health, they inevitably depend on the environmental context, such as WiFi accessibility and education or socioeconomic factors. However, they can be further influenced by age and level of computer self-efficacy [18].

**Fig. 1:**
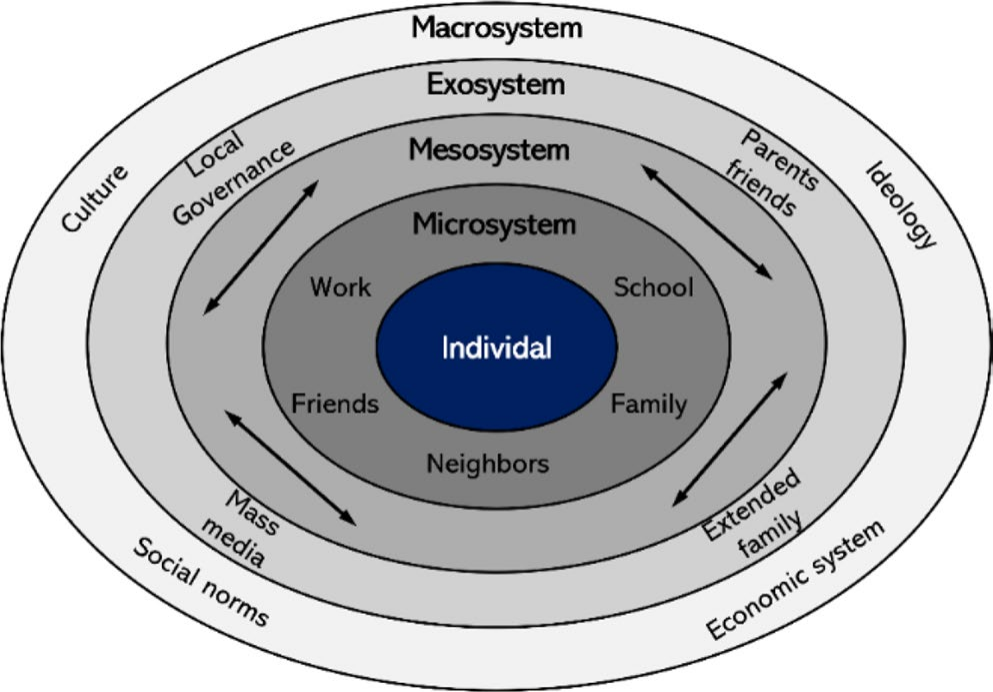
Bronfenbrenner’s Ecological Model [15]

Bronfenbrenner’s ecological model’s basis resides in environmental influences that put the individual at the innermost nested level and expand outward toward larger social systems of influence. The first level of influence involves microsystems. Microsystems include interpersonal interactions among family members, friends, teachers, and colleagues. The second level of influence, mesosystems, comprises the relationships and processes between two or more microsystems, such as interactions between home and school, peer groups and home, and work and home [16]. The next level of influence, ecosystems, is the more extensive social system that comprises two or more settings, including direct and indirect components (e.g., politics, economics, and culture). The final level, macrosystems, consists of overarching cultural and subcultural characteristics that influence all other levels, such as belief systems, knowledge, resources, and lifestyle factors (Fig. 1).

Research that applies the ecological model in the context of sport illustrates how different systems in Bronfenbrenner’s ecological model influence an individual’s participation in physical activities. For instance, a study by Zheng et al. [19] found that health inequality exists among older adults in China. More specifically, the lower the income and the worse the community-built environment, the worse the health. The community-built environment plays a vital role in the health of older adults. Moreover, the community-built environment influenced the health of older adults through the intermediary role of outdoor activities and social participation. Kim [10, 11] examined that barriers to physical activity among older people include chronic diseases, a lack of support from social significance such as family members, friends, and colleagues, a lack of sports facilities, and a lack of opportunities to participate in physical activities. These barriers can be categorized into different systems in the ecological model. Backonja et al. [20] asserted that participation in physical activity and intention to continue physical activity among older people involves a wide range of stakeholders: older adults and their families and friends, communities, health services agencies, and government agencies. These involved match the different systems in the ecological model: individual, microsystem, mesosystem, exosystem, and macrosystem. Individuals within each system and in other systems are interconnected. These connections can represent flows of information from local and/or central government agencies (exosystem and macrosystem) about how older adults (individuals) can participate in physical activity and how they create a social network of individuals (mesosystem and microsystem) who share information in choosing a different physical activity.

Bronfenbrenner’s ecological model is well-suited to understand older adults’ behaviors and attitudes toward participation in physical activities in a super-aging society like Korea. Understanding older people and environmental level factors, interactions, and limitations can guide researchers in designing applications that meet specific personal needs. The study provides a better understanding of ecological systems that affect the physical activity level of older adults by sex and, in turn, contributes to establishing policies on the health and welfare of older adults.

## Materials and methods

### Data

Data were collected using face-to-face and non-face-to-face surveys through convenience sampling from older adults aged 65 or over who actively participated in physical activities such as jogging, walking, aerobics, and swimming, at senior welfare centers, sports centers, and senior sports clubs in Korea. Only those who engaged in these activities at least once per week were included. The survey comprised 64 questions related to personal characteristics and ecological systems. A total of 537 pieces of data were collected and analyzed. The participant’s characteristics are listed in Table 1.

**Table 1:** Participants’ characteristics

	Division	Cases	Percent (%)		Division	Cases	Percent (%)
Sex	Male	249	46.4	Smoking	Yes	290	46.9
	Female	288	53.6		No	328	53.1
Age	60s	475	88.5	Drinking	Yes	364	67.8
	70s	57	10.6		No	173	32.2
	80 years and older	5	0.9				
Education	Elementary school	1	0.2	Housing type	Detached house	44	8.2
	Middle school	14	2.6		Apartment	401	74.7
	High school	148	27.6		Multiplex housing	83	15.5
	College	305	56.8				
	Graduate school or higher	69	12.8		Other	9	1.7
Family type	Single family	69	12.8				
	With spouse and children	197	36.7				
	With spouse	226	42.1				
	With children	45	8.4				

The sex distribution of the participants was 249 men (46.4%) and 288 women (53.6%), and the average age was 65.4 years. The number of participants for specific age groups was as follows: 475 (88.5%)—those in their 60s; 57 (10.6%)—those in their 70s; 5 (0.9%)—those aged 80 years and above. Regarding education level, one (0.2%) had graduated from elementary school, 14 (2.6%) completed middle school, 148 (27.6%) had graduated high school, 305 (56.8%) had graduated from university, and 69 (12.8%) had graduated school or higher. Regarding family structure, 69 (12.8%) lived in single-person households, 197 (36.7%) shared their residence with their spouses and children, 226 (42.1%) lived with only their spouses, and 45 (8.4%) were part of their children’s households. 290 (46.7%) and 364 (67.8%) are smoking and drinking, while 328 (53.1%) and 173 (32.2%) are non-smokers and non-drinkers, respectively. In terms of housing type, detached houses comprised 44 (8.2%), apartments 401 (74.7%), multiplex housing 83 (15.5%) and other 9 (1.7%).

### Variables

This study measured participants’ personal characteristics and ecological system factors. Personal characteristics included sex, age, education level, smoking status, drinking status, family type, and housing type. Ecological system factors comprised 54 items across various domains: goal content for exercise (individual system), social support and social relationships (microsystem), interactions between microsystems (mesosystem), the sports environment (exosystem), and components of the macrosystem, including an age-friendly environment and collectivism. To enhance the validity and reliability of each variable, exploratory factor analysis and reliability analysis were conducted. Factors with Eigenvalues exceeding one was retained and Internal consistency was acceptable when Cronbach’s alpha was > 0.7; a score > 0.90 was considered to be excellent.

Within the individual factor of ecological systems, the physical activity scale (Goal Content for Exercise) was used by modifying and supplementing the scale developed by Sebire [21] to suit the purpose of the study. Consequently, a total of four sub-factors and 15 questions were derived as follows: social cognition (3.473), four questions; healthcare (2.871), four questions; social belonging (3.207), four questions; and skill development (2.456), three questions. This scale was found to be reliable in terms of social cognition (α = 0.941), healthcare (α = 0.864), social belonging (α = 0.941), and skill development (α = 0.896). In the microsystem, the Social Support Scale, originally developed by Sallis et al. [22] and adapted for Korea by Choi [23], was used. This scale includes one factor and six items (4.265), demonstrating strong reliability (α = 0.917). The Social Relationship Scale, initially developed by Weiss [24] and adapted by Kim [25] for Korea, was also utilized. It comprises one factor and ten items focused on social activities (6.175) with high reliability (α = 0.927). For the exosystem, the Sports Environment Scale, created by Stahl et al. [26] and adapted for Korea by Yang et al. [27], was applied in a modified form. This scale includes one factor and six items concerning the sports environment (3.637), with reliability (α = 0.868). In the macrosystem, the Age-Friendly Environment Scale, based on the WHO’s age-friendly city guidelines [5], and was developed by Kim [28], Chung and Park [29], and Chung and Jeong [30]. This scale consists of three factors and ten items: five items on the social environment (3.356), two on the service environment (health and community support services) (1.800), and three on the physical environment (2.097). Reliability of the social and cultural environment (α = 0.880), service environment (α = 0.863), and physical environment (α = 0.747) was confirmed. Additionally, as another macrosystem factor, the Collectivism Scale, developed by Triandis and Gelfand [31] and adapted by Seon [32] for Korea, was used. This scale includes ten items measuring collectivist values (4.796) and exhibits strong reliability (α = 0.875).

### Data analysis

We analyzed the results using statistical methods. Frequency, correlation, and regression analyses were performed using SPSS (WIN 24.0, IBM, New York, US). We conducted a frequency analysis to evaluate participants’ characteristics. Subsequently, factor and reliability analyses were performed to confirm whether the variables were appropriate for the study, and a correlation analysis and regression analysis were conducted to determine the difference in ecological systems influencing physical activity between older men and women.

### Ethical considerations

This research examines ecological systems influencing the physical activity of older men and women, respectively. First and foremost, this study was approved by the Institutional Review Board of Gachon University (No. 2023-020, 22 Mar 2023). Researchers obtained informed consent from all participants before conducting research. We provided detailed information about the study’s purposes, procedures, potential risks, and benefits, allowing participants to make an informed decision about participation. Researchers allowed participants to express their right to withdraw from this study at any time. Researchers took steps to protect the privacy and confidentiality of participants’ personal information. Data were stored securely and accessed only by authorized personnel. Participants were assured that their data would be anonymized in research publications to protect their identity. Researchers treated participants with respect and dignity throughout the study process, providing appropriate support services if they experienced distress or discomfort during the study.

## Results

### Results of correlation analysis between sub-factors of ecological system

Table 2 shows the correlation analysis results between the ecological system’s subfactors. Specifically, goal content for exercise (social recognition, social affiliation, health management, and skill development), social support, social relationships, sports environment, collectivism, and age-friendly environments (social environment, physical environment, health, and community support services) were correlated.

**Table 2:** Results of correlation analysis between ecological system factors

	1	2	3	4	5	6	7	8	9	10	11
1	1.000										
2	0.305**	1.000									
3	0.025	0.366**	1.000								
4	0.559**	0.350**	0.115**	1.000							
5	0.111*	0.265**	0.296**	0.101*	1.000						
6	0.121**	0.486**	0.295**	0.225**	0.231**	1.000					
7	0.025	0.370**	0.336**	0.138**	0.241**	0.369**	1.000				
8	0.041	0.320**	0.302**	0.116**	0.197**	0.473**	0.365**	1.000			
9	0.148**	0.295**	0.125**	0.187**	0.179**	0.256**	0.498**	0.235**	1.000		
10	0.034	0.308**	0.371**	0.049	0.213**	0.288**	0.480**	0.295**	0.391**	1.000	
11	0.087*	0.261**	0.244**	0.128**	0.148**	0.269**	0.450**	0.286**	0.501**	0.427**	1.000

1 = social recognition, 2 = social affiliation, 3 = health management, 4 = skill development, 5 = social support, 6 = social relationship, 7 = sports environment, 8 = collectivism, 9 = social environment, 10 = physical environment, 11 = health and community support service

### Analysis of ecological systems influencing physical activity participation in older males

Regression analysis was conducted to determine the mutual influence between ecological systems targeting the older male group aged 65 years or above in Korea. The results are summarized in Table 3. First, macrosystem influenced exosystem (β = 0.614, *p* <.001) and microsystem (β = 0.389, *p* <.001). Second, the exosystem affected the mesosystem (β = 0.235, *p* <.01), and third, the microsystem was found to affect the individuals (β = 0.287, *p* <.001).

**Table 3:** Regression analysis from macrosystem to individual (Male group)

			Estimate(Β)	S.E.	C.*R*.	*p*	Estimate(β)
Macro	>	Exo	0.986	0.081	12.252	***	0.614
	>	Meso	− 0.038	0.056	− 0.688	0.492	− 0.054
	>	Micro	0.459	0.082	5.596	***	0.389
	>	Individual	0.191	0.103	1.845	0.065	0.143
Exo	>	Meso	0.103	0.035	2.986	**	0.235
	>	Micro	0.096	0.052	1.857	0.063	0.131
	>	Individual	0.059	0.062	0.956	0.339	0.072
Meso	>	Micro	0.179	0.094	1.916	0.055	0.107
	>	Individual	0.007	0.112	0.066	0.947	0.004
Micro	>	Individual	0.323	0.075	4.289	***	0.287

*p <.05, **p <.01, ***p <.001

### Analysis of ecological systems influencing physical activity participation in older females

**Fig. 2:**
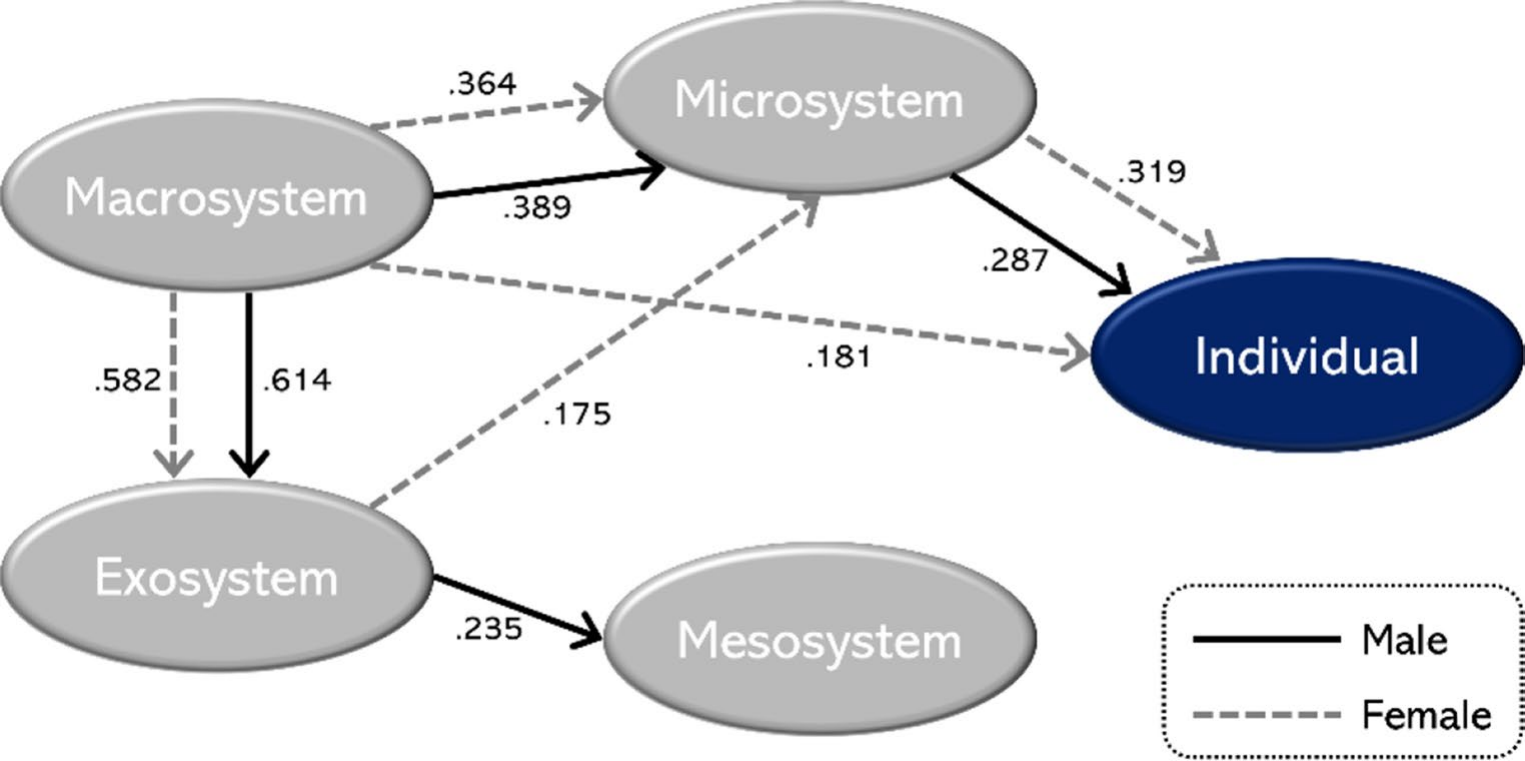
Differences in Ecological Systems Influencing Physical Activity between Older Men and Women

A regression analysis was conducted to investigate ecological systems’ influence on older females aged 65 years or above in Korea. The results are summarized in Table 4; Fig. 2. First, the macrosystem affected the exosystem (β = 0.582, *p* <.001), microsystem (β = 0.364, *p* <.001), and individuals (β =. 181, *p* <.01). The exosystem was found to have a significant effect on the microsystem (β = 0.175, *p* <.01), and the microsystem was found to have a significant effect on individuals (β = 0.319, *p* <.001).

**Table 4:** Regression analysis from macrosystem to individual (Female group)

			Estimate(Β)	S.E.	C.*R*.	*p*	Estimate(β)
Macro	>	Exo	0.787	0.065	12.127	***	0.582
	>	Meso	− 0.101	0.057	-1.776	0.076	− 0.128
	>	Micro	0.487	0.085	5.713	***	0.364
	>	Individual	0.205	0.077	2.665	**	0.181
Exo	>	Meso	0.080	0.042	1.918	0.055	0.138
	>	Micro	0.173	0.063	2.739	**	0.175
	>	Individual	0.055	0.055	1.002	0.316	0.065
Meso	>	Micro	0.011	0.088	0.126	0.900	0.007
	>	Individual	− 0.039	0.075	− 0.519	0.604	− 0.027
Micro	>	Individual	0.270	0.050	5.355	***	0.319

*p <.05, **p <.01, ***p <.001

As seen in Fig. 2, we examined the ecological systems that affect physical activity of older men and found that the macrosystem affects the exosystem and microsystem, the exosystem affects the mesosystem, and the microsystem affects individuals. For women, the macrosystem affects the exosystem, microsystem, and individuals; the exosystem affects the microsystem, and the microsystem affects individuals.

## Discussion

This study aimed to examine the ecological systems that affect older men’s and women’s physical activity and to identify differences by sex. Specifically, we determined whether the ecological systems influencing the periodic physical activity of older adults vary by sex. Of the responses collected through the online and offline surveys, 537 were used for analysis, with a regression analysis performed for each sex.

According to Bronfenbrenner’s ecological model [15], an individual’s development is influenced by a series of interconnected ecological systems, ranging from the surrounding environment to broader social structures. In other words, the physical activity level of older adults is determined by various environmental factors. The results of this study indicate the complex interactions between the different ecological systems.

A particularly noteworthy finding is the importance of the microsystem. The microsystem directly influenced older men’s and women’s physical activity levels. This result can be interpreted as a positive impact of social support on the physical activity of older men and women. The results of previous studies support the findings in this study [11, 14, 33–35]. For example, according to a study by Kim [11], who explored strategies to promote physical activity among older adults living in small- and medium-sized cities, a lack of support from people around them led to a decline in their physical activity. This is because people around older adults such as family members and friends are essential information providers with whom they can engage in physical activities [11].

These results have implications for the importance of significant others who directly encourage and support older adults’ physical activities. For older adults, significant others may be a spouse, child or friend, and those living alone may be neighbors. In other words, it refers to the individual that most closely influences their life, and the signifcant others’ interests can lead to an increase in their physical activities. Bronfenbrenner also initially emphasized the importance of the macrosystem but later further emphasized the microsystem, which can directly affect individuals [36].

As the role of significant others becomes increasingly essential, institutional efforts are required to support them. Indeed, Korea has established various policies, such as introducing Long-term Care Insurance and increasing community nursing and medical facilities [37], but these policies are unstable. Long-term Care Insurance reserves are expected to be depleted by 2026, and the system needs to be improved because of the limited number of people receiving care and the low quality of services [38]. In addition, older adults can engage in physical activity at home with their significant others depending on their health level; therefore, it is necessary to provide systematic exercise methods and welfare information to guardians. In addition, alternative measures, such as care for older adults, should be provided more reliably to those without significant others.

Furthermore, while the macrosystem affects older women, it does not affect older men. Based on these results, we could more clearly analyze the causes of low physical activity in older men. Older individuals mainly participate in exercise programs provided by public sports facilities or community welfare centers with low financial burdens [3]. These programs actively promote physical activity among older adults and are operated through government or local government budgets. In other words, as this is a public policy, it requires no personal costs and is easily accessible.

These efforts are part of the macrosystem, including institutions and cultures. Why does the macrosystem, essential for the older adult’s physical activity, affect only women? Senior citizen sports policies seem less influential in helping older men engage in physical activity. According to a study by Yun and Choi [39], the participation rate of older men in exercise programs conducted at senior welfare centers was very low. Most exercise programs are conducted in groups; however, in Korea, the participation rate of older men in programs in which several people exercise together is very low [28].

While the causes of this phenomenon have been extensively analyzed, the cause that has been repeatedly raised is the organization of exercise programs focusing on sports favored by older women [39–41]. Community exercise programs for older adults mainly include aerobics, yoga, and silver dancing, which limit the participation of older men [39–41]. However, solving this problem is difficult because of complex institutional and cultural limitations.

Exercise programs require institutional improvements to expand opportunities for older men. However, this is more than just a system that requires improvement. According to Yun and Choi [39], a culture of reluctance to take classes in which many women participate due to social stereotypes about men limits older men’s physical activity. In other words, the unique patriarchal tendencies of Korean men make them hesitant to participate in exercise programs in which many women participate. In particular, the current older generation has preferred patriarchal and conservative masculinity. Therefore, it is difficult to adapt to a group with more women than men, and the tendency to be aware of societal views is prominent.

Therefore, the overall supplementation of the macrosystem is necessary to promote participation in physical activities among older men. This should be followed by institutional and cultural efforts. Institutionally, expanding public sports facilities and providing economic support will ensure older adults have access to a wide variety of exercise programs. For example, certain activities are widely perceived as gender-neutral and may appeal to older men, such as hiking, walking clubs, rock climbing, and swimming. These activities provide inclusive options without strong gender associations and could serve as effective tools for promoting participation across genders. Structured community support networks, such as men’s health groups or peer-led fitness programs, could further foster a supportive environment, reducing any reluctance they may feel toward participation in these activities.

Additionally, identifying specific sports that older men prefer and ensuring that community spaces are equipped with skilled professionals can help attract and sustain interest. However, cultural efforts are also essential. The community—individuals, local leaders, and institutions—must work toward dismantling patriarchal stereotypes and cultivating a culture that actively encourages older adults to participate in physical activities. Moving beyond gender-specific programs, communities could promote sports without gender stereotypes allowing men and women to exercise together and fostering a more inclusive environment. These initiatives, along with culturally sensitive strategies, offer a pathway for engaging older men in physical activity in a way that is both supportive and sustainable.

Finally, comparing these findings to other countries highlights the influence of cultural norms on participation [42, 43]. In cultures with more flexible gender roles, older men may face fewer barriers to engaging in a broader range of activities. For instance, in Western countries where gender norms are generally less rigid, older men might more readily participate in community exercise programs regardless of gender composition, suggesting that cultural norms play a substantial role in shaping activity preferences and participation rates. These comparisons underscore the need for culturally sensitive strategies to increase physical activity participation among older adults.

## Conclusion and recommendations

This study examined the ecological factors influencing physical activity levels among older adults, considering gender differences within the framework of Bronfenbrenner’s ecological model. Through this approach, we aimed to shed light on how personal, social, environmental, and cultural factors interact to shape physical activity behaviors in older men and women.

First, by examining the ecological systems that affect the physical activity of older men, we found that the macrosystem affected the exosystem and microsystem, the exosystem affected the mesosystem, and the microsystem affected individuals. Second, in the case of women, the macrosystem affected the exosystem, microsystem, and individuals; the exosystem affected the microsystem, and the microsystem affected individuals. In summary, the microsystem commonly affects the physical activity of older men and women, whereas the macrosystem only affects the physical activity of older women. These results suggest that older men do not receive help from the macrosystem.

To address this disparity, community initiatives should broaden the range of exercise programs and promote a supportive social atmosphere that encourages gender-inclusive sports participation. Policymakers and community leaders could use these findings to design targeted programs and policies that enhance physical activity participation among older men, thereby supporting equitable health outcomes across genders.

Furthermore, future research directions should expand on longitudinal studies to assess changes in physical activity patterns over time, providing deeper understanding of causative relationships among ecological factors. Addressing the study’s limitation related to self-reported data, future studies could incorporate objective physical activity measurements to enhance data accuracy. Enlarging and diversifying the sample would also improve the generalizability of findings, providing more robust insights into the ecological determinants of physical activity for older adults across various demographic groups.

## Data Availability

No datasets were generated or analysed during the current study.
